# Evaluation of the Effects of Nanoparticle Mixtures on Brassica Seed Germination and Bacterial Bioluminescence Activity Based on the Theory of Probability

**DOI:** 10.3390/nano7100344

**Published:** 2017-10-23

**Authors:** Kyung-Seok Ko, Dong-Chan Koh, In Chul Kong

**Affiliations:** 1 Geologic Environment Division, Korea Institute of Geoscience & Mineral Resources (KIGAM), Daejeon 34132, Korea; kyongsok@kigam.re.kr (K.-S.K.); chankoh@kigam.re.kr (D.-C.K.); 2 Department of Mineral & Groundwater Resources, University of Science and Technology, Daejeon 34113, Korea; 3 Department of Environmental Engineering, Yeungnam University, Kyungbuk 38541, Korea

**Keywords:** binary mixture, bioluminescence, nanoparticles, probability, seed germination, toxicity

## Abstract

Effects of binary mixtures of six metal oxide nanoparticles (NPs; 54 combinations) on the activities of seed germination and bacterial bioluminescence were investigated using the theory of probability. The observed toxicities of various NPs combinations were compared with the theoretically expected toxicities, calculated based on individual NPs toxicities. Different sensitivities were observed depending on the concentrations and the types of NPs. The synergistic mode (67%; observed toxicity greater than expected toxicity) was predominantly observed in the bioluminescence test, whereas both synergistic (47%) and additive (50%) modes were prevalent in the activity of seed germination. With regard to overall analysis, a slightly high percentage (56%) of the synergistic mode of action was (30 out of 54 binary mixture combinations; *p* < 0.0392) observed. These results suggest that the exposure of an NPs mixture in the environment may lead to a similar or higher toxicity level than the sum of its constituent NPs would suggest. In addition, one organism for assessment did not always show same results as those from a different assessment. Therefore, combining results of different organisms exposed to a wide range of concentrations of binary mixture will more properly predict and evaluate the expected ecotoxicity of pollutants on environments.

## 1. Introduction

These days, many nanoparticles (NPs) products are available in the areas of textiles, electronics, medical devices, cosmetics, environmental treatment processes, etc. [1]. The use and disposal of NPs in these fields will lead to intentional or accidental release of NPs into the environment [2]. NPs are generally classified into carbon-based, metal-based, dendrimers, and composite materials [3]. The detection and quantification of NPs are very difficult tasks in complex environmental systems [4].

Many studies on NPs toxicity demonstrate different levels of toxicity for various types of NPs [5,6]. Metal- and carbon-based NPs are relatively common and are frequently studied. Some studies reported the toxicity of TiO_2_ and ZnO NPs to crustaceans, microalgae, and bacteria [7,8]. Regarding the NPs of Ag, Pt, and carbon nanotubes, several reports have been published on the toxicity to bacteria or terrestrial animals [9,10]. The understandings of the toxicity mechanism varied depending on the specific NPs and with the study; therefore, toxicological effects of NPs may depend on the test method and their specific properties, such as size, surface characteristics, reactivity, optical sensitivity, etc. [11,12,13,14,15].

The choice of a proper test organism, endpoint, or the test organism’s sensitivity are important in the investigation of the ecotoxicity of contaminants. Toxicity tests using various plants are particularly relevant in the case of toxic contaminants in soil [16]. These have generally been studies with plant processes of seed germination and root/shoot growth. These studies are some of the simplest acute methods used in environmental monitoring [17]. More studies should investigate the toxicity of NPs on various ranges of response endpoints of plants (e.g., biomass, enzyme activity etc.) with respect to the particle size, uptake, rhizosphere, and root surfaces because one species and endpoint cannot fully evaluate the toxicity of NPs on the plants [18]. Bioluminescence assays are also widely used as an appropriate sensitive method to determine the acute toxicities of various sample types and have several advantages (e.g., short exposure times, convenient signal measurements, cost efficiency, etc.) over other techniques [19,20].

Studies investigating mixture effects, rather than individual effects, present a more realistic reflection due to the exposure of complex mixtures of contaminants in environment. The evaluation of mixture effects is the difficult tasks in environmental assessment [21]. However, most toxicity studies have generally evaluated the effect of single contaminants under laboratory controlled conditions [22]. In general, mixture models can be used to solve the problems associated with this assessment. Concentration and response (effects) addition models are two basic types of mixture models [23,24]. In addition, the theoretically expected effects of mixtures can be evaluated using a simple mathematical model based on the theory of probability [25] or by using the toxic unit (TU) approach [26]. Based on the results of these evaluation, the mixture effect can be classified into one of three categories: similar to (additive), greater than (synergistic), or less than (antagonistic), expected effects calculated theoretically based on individual chemicals. The adoption of a specific model is based on the characteristics of the action of each pollutant [22].

The objective of this research was to evaluate the modes of interactive toxic effects of metal oxide NPs (CuO, NiO, ZnO, TiO_2_, Fe_2_O_3_, and Co_3_O_4_) on seed (*Brassica*) germination and a bioluminescence-producing *E. coli* mutant strain. Interactive modes of action of binary mixtures of NPs were determined using the theory of probability.

## 2. Results

### 2.1. Interactive Effects on Bioluminescence Activity

**Figure 1:**
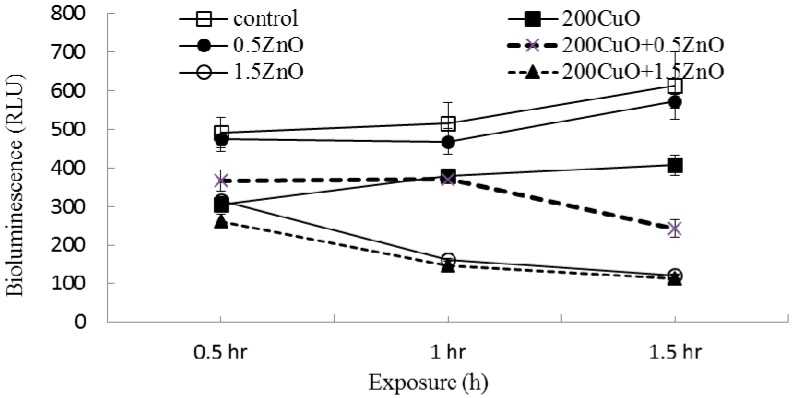
Representative results of bioluminescence activity under three individual NP treatments and two NPs mixtures. “200 CuO + 0.5 ZnO” represents a mixture of 200 mg/L CuO and 0.5 mg/L ZnO.

Effects of the binary NPs mixtures on bioluminescence activity were examined under 24 different mixture combinations of two concentrations of CuO, NiO, ZnO, and Co_3_O_4_. The controls (no NPs applied) produced bioluminescence (the sum of values observed after 1 and 1.5 h) in the range of 1130 ± 142.2–1626 ± 87.6 RLU (relative light unit). Generally, sets with all NPs showed some level of inhibition on the bioluminescence activity. Comparisons of two representative results of the bioluminescence activities of individual and mixed NPs (200 mg/L CuO mixed with 0.5 or 1.5 mg/L ZnO) are shown in Figure 1. In the presence of individual ZnO NPs, significant activity reduction was observed at 1.5 mg/L ZnO compared to 0.5 mg/L ZnO. The observed bioluminescence (the sum of values observed after 1 and 1.5 h) was 1014 and 284 RLU with 0.5 and 1.5 mg/L ZnO, respectively. A mixture of 200 mg/L CuO and 0.5 mg/L ZnO produced 613 ± 32 RLU (corresponding to 54% of control activity), whereas the individual treatments of 200 mg/L CuO and 0.5 mg/L ZnO produced 787 ± 33 RLU and 1041 ± 51 RLU (corresponding to 70% and 92% of control activity), respectively. In contrast, a mixture of 200 mg/L CuO and 1.5 mg/L ZnO resulted in a bioluminescence value of 261 RLU, which is approximately 2.4 times lower than that of a mixture of 200 mg/L CuO and 0.5 mg/L ZnO.

Information for ranges of the relative bioluminescence toxicities of individual and binary mixtures of NPs are shown in Table 1. Under the tested conditions, sets with individual NPs showed bioluminescence toxicity values ranging from 0 ± 6.4% (70 mg/L NiO) to 75 ± 0.6% (1.5 mg/L ZnO), while those with binary mixtures of NPs showed bioluminescence toxicity values from 49 ± 14.9% (200 mg/L CuO and 0.5 mg/L ZnO) to 97 ± 1.3% (1.5 mg/L ZnO and 70 mg/L NiO). Average toxicities of individual NPs and binary mixtures were 28% and 82%, respectively.

**Figure 2:**
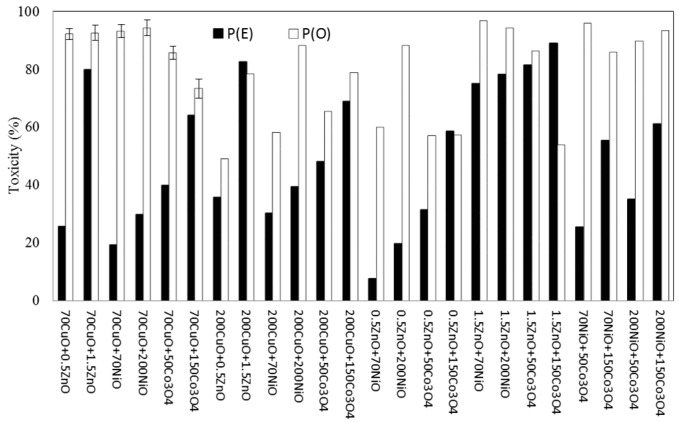
Comparison between theoretically expected and observed effects of binary NPs mixtures on bioluminescence activity of RB1436 showing synergistic mode of action. P(E), the theoretically expected inhibition; P(O), the observed inhibition of the binary mixture. “70 CuO + 0.5 ZnO” means the binary mixture of 70 mg/L CuO and 0.5 mg/L ZnO.

**Figure 3:**
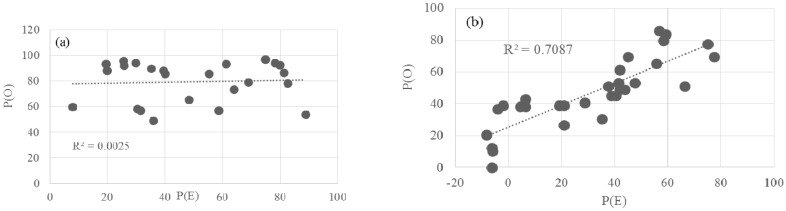
Correlations between theoretically expected and observed activities of (**a**) bacterial bioluminescence (24 combinations) and (**b**) seed germination (30 combinations) in the presence of binary mixtures of various NPs.

The observed toxic effect, P(O), of each of the 24 combinations of binary NPs mixtures was compared with its expected toxic effect, P(E), which was calculated using the theory of probability from two measurements of individual effects. Of the different binary NPs mixtures, a broad spectrum of bioluminescence toxicity, both P(E) and P(O) was observed, with ranges of 8–89% and 49–97%, respectively (Figure 2). Among the different NPs combinations, the mixture of 28 mg/L ZnO with 2000 mg/L of Fe_2_O_3_, Co_3_O_4_, or TiO_2_, showed relatively high P(O), showing in the range of 80–86% toxicity. The average toxicities of all combinations of P(E) and P(O) were 49 ± 25.2% and 80 ± 38.9%, respectively. Ratios of observed toxicity to expected toxicity (P(O)/P(E)) ranged from 0.6 to 7.6 (avg. 2.2). The pattern of correlation between P(O) and P(E) showed a very low coefficient of determination (*R*^2^ = 0.0025) (Figure 3a).

### 2.2. Interactive Effects on Seed Germination

Based on preliminary experiments in several seed species, *Brassica* seeds were chosen for evaluating the interactive NPs mixture effects on seed germination (30 different mixture combinations were used). Two concentrations (low and high) for each NPs were set based on the preliminary concentration-finding tests (Table 1). In the control (no NPs treatment), an average of 16 ± 0.6 seeds per batch out of 20 total seeds germinated successfully (successful germination was defined as growth greater than 2 cm) during the three-day incubation period. Under the tested conditions, the numbers of germinated seeds were in the ranges of 6–17 and 2–17 seeds out of 20 with individual NPs and binary mixtures, respectively. Sets with individual NPs showed seed germination toxicity values ranging from −6% (2000 mg/L Co_3_O_4_; stimulation) to 59% (28 mg/L ZnO), while those with binary mixtures of NPs showed seed germination toxicity values ranging from 0% (1000 mg/L Co_3_O_4_ and 1000 mg/L TiO_2_) to 86% (28 mg/L ZnO and 1000 mg/L Co_3_O_4_). The average toxicities of individual NPs and binary mixtures were 17% and 47%, respectively (Table 2).

**Figure 4:**
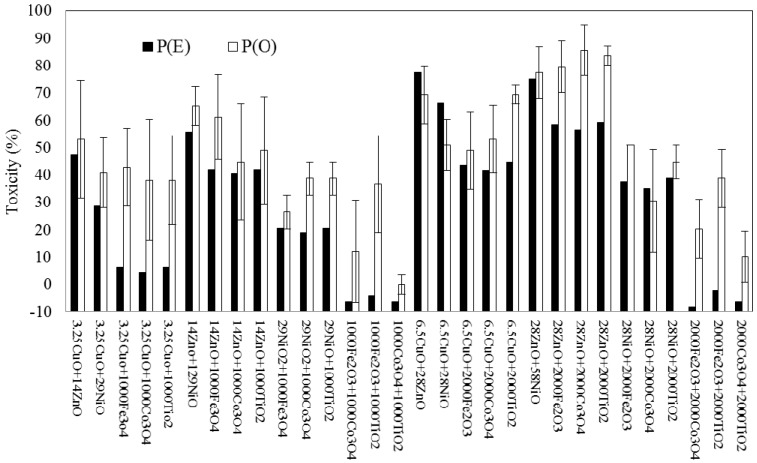
Comparison between theoretically expected and observed effects of binary NPs mixtures on the activity of seed germination showing synergistic and additive modes of action. P(E), the theoretically expected inhibition; P(O), the observed inhibition of the binary mixture. “14 ZnO + 1000 Co_3_O_4_” means the binary mixture of 14 mg/L ZnO and 1000 mg/L Co_3_O_4_.

With the different binary NPs mixtures, wide ranges of seed germination toxicity were observed, showing −8% to 78% and 0% to 86% for P(O) and P(E), respectively (Figure 4). Among the different NPs combinations, the highest observed toxicity value on seed germination was 86 ± 9.4% for the mixture of 28 mg/L ZnO and 2000 mg/L Co_3_O_4_. The average toxicities of all combinations of P(E) and P(O) were 31 ± 26.0% and 47 ± 21.3%, respectively. Ratios of observed toxicity to expected toxicity (P(O)/P(E)) were ranged from 0.8 to 8.7 (avg. 2.7). The pattern of correlation between P(O) and P(E) also showed a coefficient *R*^2^ = 0.7087, which is much higher than that found for bacterial bioluminescence (Figure 3b).

## 3. Discussion

The effects of contaminants on the environment should be assessed using various types and concentrations of chemical mixtures as well as different test organisms. Based on initial definitive test, different concentrations of each NPs were determined for the investigation of the binary mixture effects from the minimum of 0.5 (ZnO) mg/L to the maximum of 2000 (Co_3_O_4_, Fe_2_O_3_, TiO_2_) mg/L, depending on the NPs (Table 1). Among the six tested NPs, ZnO was the most toxic based on the bacterial bioluminescence activity test, whereas CuO was the most toxic for seed germination. In general, most of the tested concentrations of individual NPs and their binary mixtures inhibited bacterial bioluminescence and seed germination under the experimental conditions used. However, there were a few cases where mild stimulation of seed germination was observed, especially in the presence of metal oxides of Co, Ti, and Fe. For example, slight stimulation of seed germination (2–6% stimulation) was observed at 1000 and 2000 mg/L of Ti, Co, and Fe metal oxide NPs.

Bacterial bioluminescence is widely used as a time-efficient, cost-effective, and sensitive method for assessing the effect of pollutants on environmental samples [19]. A binary mixture may have one of the following three distinct effects: synergistic (greater than additive), additive, and antagonistic (less than additive) [27]. In this investigation, the observed bioluminescence toxicity, P(O), of the binary NPs mixtures was between 49 ± 14.9% and 97 ± 1.7% (avg. 82 ± 14.5%), whereas the expected toxicity, P(E), was in the range of 8–89% (avg. 49 ± 23.3%), determined based on the theory of probability. According to the statistical significance analysis of P(O) and P(E) of each combination in the bioluminescence test, mostly additive and synergistic modes of action were observed with the binary mixtures, showing 8 (33%; *p* > 0.0821) and 16 (67%; *p* < 0.0091) out of 24 combinations, respectively. However, the average toxicities of all combinations of P(E) and P(O) were 49 ± 23.3% and 82 ± 14.5%, respectively (statistically significant differences; *p*-value 0.0001). Therefore, in terms of overall results, synergistic toxicity (observed toxicity being greater than expected toxicity) was the main mode of action when tested by assessing the effect of NPs mixtures on bacterial bioluminescence activity. The ratios also ranged from 0.6 to 5.3 (avg. 2.1), indicating that the observed toxicities were generally greater than the expected toxicities.

Although the precise toxicity mechanisms of most NPs are clearly unknown, researchers have reported that toxicity is generally affected by shape, particle size, and surface properties such as a positive charge [13,14]. Studies also have reported that the effects of NPs on bacteria could occur through membrane disruption, surface photocatalytic oxidation, DNA damage, and reactive oxygen species (ROS) production, etc. [28,29,30,31,32]. Studies reported that ZnO NPs can induce the production of ROS, which can cause membrane damage by affecting lipids, carbohydrates, and proteins that constitute it; pits in the membrane, leading to increased membrane permeability and cell death, can also be created due to their small size [33,34,35]. Dissolved metal ions from NPs also may be responsible for the antibacterial effects, as bacteria are mostly protected against NPs transport into cytoplasm for colloids [7]. Tong et al. [36] and Marambio-Jones and Hoek [37] reported that the toxicity of Ag NPs is mainly caused by the free ions. In our previous investigation, metal ions released from the NPs in soil slurry was less than 6% of the added NPs concentration and these metal ion concentrations induced less than 25% toxicity when tested using bacterial bioluminescence [38]. Therefore, the toxicity of NPs might be induced not only by released ions but also by the particle characteristics; this will depend on the test conditions.

Several plant species can serve as indicators for determining the toxicity of soil contamination, responding rapidly to the toxic effects of chemicals. Liu et al. [39] reported that seed germination is one of the well-known indicators among other endpoints of root length, shoot height, root, shoot, or total biomass. In the present study, the test using seed germination showed slightly different outcomes than the bioluminescence test. Unlike the bioluminescence activity, which showed highly synergistic modes of action, the seed germination test yielded similar numbers of NPs exhibiting synergistic and additive patterns of interactive modes, with 14 (47%; *p* < 0.0392) and 15 (50%; *p* > 0.0568) out of 30 mixture combinations, respectively. With respect to average toxicities, statistically significant additive modes of action were observed (*p* value 0.4692) between P(O) (47 ± 21.3%) and P(E) (31 ± 26.0%). Among 30 mixture combinations, only one antagonistic toxic effect on seed germination was observed for the mixture of 6.5 mg/L CuO and 28 mg/L NiO, with 66% and 51% toxicity for P(E) and P(O), respectively (*p* = 0.0471). Therefore, in terms of overall results, additive toxicity (observed toxicity being similar to expected toxicity) was the main mode of action when tested by assessing the effect of NPs mixtures on seed germination activity. The ratios of observed toxicity to expected toxicity (P(O)/P(E)) was ranged from 0.6 to 5.3 (avg. 2.7), indicating that the observed toxicities were generally greater than the expected toxicities.

The effects of NPs on seed germination are dependent on their ability to reach embryonic tissues across the seed coat [39,40]. This ability is mainly dependent on the seed coat structure of each plant species and changes according to the physical and chemical properties of the environmental pollutants [41]. The inhibition of specific enzymatic reactions by NPs to various enzymes such as amylase could also explain NPs toxicity on seed germination. Different NPs, depending on their size and type, may undergo unique patterns of agglomeration, sorption, desorption, and dissolution, which could be essential to NPs toxicity [42]. In the present study, considerable adsorption of Co and Ti oxide NPs was observed compared to other tested NPs. Such characteristics could also influence on the activity of organisms. It is not clear at this point whether NPs toxicity is induced by the solubilized ions or by the particles themselves.

Kungolos et al. [25] reported that the interactive type of action might depend not only on the type of chemicals in the mixture but also their relative concentrations. In our previous investigation using binary metal mixtures, an additive mode of action was mostly observed in the bioluminescence assay, whereas additive and synergistic modes were mostly observed in seed germination [43]. With regard to binary NPs mixtures overall, the synergistic mode of action was predominant, with 30 (56%) out of 54 binary mixture combinations (*p* < 0.0392). It was followed by the additive mode of toxicity (23 combinations; 43%). This synergistic phenomenon could be due to the more bioavailable chemical mixtures and to the interactions that increase the bioavailability of mixture compounds in the medium, thereby increasing chemical uptake [44,45].

## 4. Materials and Methods

### 4.1. Chemicals and Preparation

Six types of metal oxide NPs were tested in this study: TiO_2_ (<25 nm), Fe_2_O_3_ (20–40 nm), CuO (30–50 nm), NiO (30 nm), and Co_3_O_4_ (10–30 nm) (obtained from Nanostructured and Amorphous Materials, Houston, TX, USA), ZnO (40–100 nm) (obtained from Alfa Aesar, Tewksbury, MA, USA). The NPs suspended directly in deionized water were dispersed by ultrasonic vibration for 10 min prior to use. All other chemicals were reagent-grade and purchased from Sigma and Aldrich (St. Louis, MO, USA).

### 4.2. Effect of NPs on Bioluminescence Activity

The acute toxicities of the NPs were evaluated using a mutant strain of *Escherichia coli* DH5ɑ RB1436 (called RB1436; obtained from Dr. R. S. Burlage of Concordia University, Mequon, WI, USA). This strain contains a spontaneously deleted pUCD615 plasmid and produce bioluminescence (luminescent light) during its growth periods [46].

The bacteria were cultured overnight in Luria-Bertani^ka^ (LB^ka^) medium (tryptone 10 g, yeast extract 5 g, NaCl 5 g, 2 N NaOH 0.5 mL, kanamycin 50 mg per liter) at 27 °C with shaking (130 rpm). Following subsequent dilution (1:30 in LB^ka^ medium), the cultures was cultivated until an optical density (OD_600_) of approximately 0.6. For the toxicity test, 9 mL of the sample was mixed with 1 mL of the bacterial culture (OD_600_ of 0.2) and incubated. Bioluminescence was measured with a Turner 20/20 luminometer (Turner Design Inc., Sunnyvale, CA, USA), where the maximum detection limit was 9999 relative light units (RLU).

### 4.3. Effect of NPs on Seed Germination

Seeds (*Brassica rapa* var. *glabra Regel*) produced by a seed company (Seoul, Korea), were obtained from a local seed store. Seeds were sterilized in 3% H_2_O_2_ and then rinsed with distilled water prior to test. Filter paper placed on a Petri dish was moistened with 5 mL of sample solution containing NP (distilled water for the control). Each plate, containing 20 seeds, was then covered by a lid and incubated in the dark at 23 ± 2 °C. When both the plumule and radicle extended longer than 2 cm from their junction, germination was considered positive after three days of incubation. Three replicates were performed for each treatment.

### 4.4. Toxicity Evaluation and Statistical Analysis

In the mixture interactive effects test, P(E) of each binary mixture (P_1_ and P_2_ : inhibition caused by chemicals ‘1’ and ‘2’) was evaluated using a model based on the theory of probability: P(E) = P_1_ + P_2_ − (P_1_P_2_/100) [38]. P(E), calculated by the above equation was compared with the P(O), determined by the experiment. The significance of each test result (synergistic, antagonistic, or additive) was determined with respect to the theoretically predicted probability (*p*) value for that binary mixture.

The concentration ranges of the mixture combinations (24 for the bioluminescence and 30 for the seed germination assays) were determined using a preliminary concentration-finding test (Table 1).

## 5. Conclusions

In conclusion, the goal of this study to identify the possible toxic effects of NPs mixtures in two different test organisms was investigated based on the theory of probability. With respect to overall results, the synergistic mode of action was predominantly observed in the case of bioluminescence, while both synergistic and additive modes of action were observed in the case of seed germination. However, in terms of average toxicities on each method, synergistic and additive modes of action were observed in case of bioluminescence and seed germination, respectively. This indicates that the presence of multiple NPs in the environment at the same time may pose an increased risk to specific organisms. These results also indicate that the effects of NPs mixtures varied depending on the test organisms used, as well as the combination conditions and concentrations of NPs mixtures. Therefore, combining results of different methods exposed to a wide range of concentrations of binary mixture will better evaluate and predict the possible ecotoxicity in contaminated environments. Clearly, more efforts need to be carried out to prove the mechanism of action of individual NPs and their mixtures in environmental samples.

## Figures and Tables

**Table 1:** Two concentrations of each nanoparticle (mg/L) used in various combinations to create binary NPs mixtures for the bioassays

Activity	CuO	ZnO	NiO	Co_3_O_4_	Fe_2_O_3_	TiO_2_	Combinations
Bioluminescence	70, 200	0.5, 1.5	70, 200	50, 150	–	–	24
Seed germination	3.25, 6.5	14, 28	29, 58	1000, 2000	1000, 2000	1000, 2000	30

**Table 2:** Relative toxicity ranges of individual nanoparticles (NPs) and binary mixtures of NPs, as assessed by (**a**) bioluminescence activity and (**b**) seed germination

Assays	Ranges of Relative Toxicity (%)	
	Individual Sets	Binary Mixture Sets
Bioluminescence	0% to 75%	49% to 95%
*Brassica* germination	−6% to 59%	14% to 102%
